# Investigating the evidence for effective digital skin surveillance methods

**DOI:** 10.1093/occmed/kqag028

**Published:** 2026-06-02

**Authors:** F Fabbri, I Albusaidi, G Ntani, K Alden, V Parsons

**Affiliations:** Occupational Health, Safety and Wellbeing Service, Guy’s and St Thomas NHS Foundation Trust, London, London, SE1 7NJ, UK; Occupational Health, Safety and Wellbeing Service, Guy’s and St Thomas NHS Foundation Trust, London, London, SE1 7NJ, UK; MRC Lifecourse Epidemiology Unit, University of Southampton, Southampton, SO16 6YD, UK; London Centre for Work and Health, London, SE1 7NJ, UK; London Centre for Work and Health, London, SE1 7NJ, UK; Occupational Health, Safety and Wellbeing Service, Guy’s and St Thomas NHS Foundation Trust, London, London, SE1 7NJ, UK; London Centre for Work and Health, London, SE1 7NJ, UK

## Abstract

**Background:**

Hand dermatitis is a common occupational skin disease and is particularly prevalent among healthcare workers.

**Aims:**

To identify which digital skin surveillance methods are effective for detecting hand dermatitis.

**Methods:**

A scoping review. Protocol, including search strategy, was developed using the PICO framework and the PRISMA-Scoping Review guide.

**Results:**

Three databases were searched with the following outputs: MEDLINE (*n* = 150), CINAHL (*n* = 568), EMBASE (*n* = 30) along with Cochrane Work (*n* = 0) and the grey literature. Twenty-eight duplicates were removed. Following screening title, abstract and full-text stages, five articles were included. Across the included studies, different digital skin surveillance tools were developed and tested to screen for hand dermatitis, producing mixed findings. Methods included rapid self-assessment questionnaires (often with photo grading classification of dermatitis to aid assessments) compared with assessments by clinicians using hand photographs. One study demonstrated that a screening method had a high negative predictive value (91%; 95% CI 89–93) but a low positive predictive value (39%; 95% CI 34–45). While able to demonstrate acceptable discriminatory accuracy between people with and without the disease and having a high specificity (86%; 95% CI 84–88), sensitivity was modest at 52% (95% CI 46–59), meaning true cases were not identified. In another study, validity testing results of self-assessment screening compared with screening by a dermatologist using photos showed only fair agreement between the two methods, highlighting variability.

**Conclusions:**

While the findings are promising, there is a compelling case to test such methods among different at-risk workers within a workplace skin surveillance programme.

Key learning points **What is already known about this subject:** 

- In the UK, hand dermatitis is a common occupational skin disease, affecting an estimated 84 000 individuals whose condition is caused or aggravated by their work. It is especially common among healthcare workers.
- Once an individual has developed irritant contact hand dermatitis, the prognosis is poor and the disease is often not picked up until relatively late in the disease, making it harder to treat and increasing the risk of them being unable to continue to work.
- There is a statutory responsibility (COSHH Regulations 2002) for employers to carry out health surveillance for hand dermatitis; the reality of enforcing health surveillance for hand dermatitis is problematic due to logistic and economic issues; thus, the necessity to find a way to efficiently deliver a surveillance programme.

**What does this study adds:** 

- This study assessed the available published literature to determine which digital skin surveillance methods using brief self-assessment screening questionnaires in combination with hand photographs (assessed by clinicians) have been developed and tested to ascertain their effectiveness in a workplace setting.
- This study showed that while various methods have been developed and were found to be reliable for use with specific workers’ groups, wider feasibility testing (pragmatic research) involving other at-risk groups within the healthcare sector (clinical and non-clinical roles) and other high-risk worker groups is required.

**What impact may this have on practice or policy:** 

- The findings from this scoping review will inform the development of a planned study to investigate whether using a single question to screen for hand dermatitis is practical, well-received and effective in a workplace health surveillance programme in the National Health Service (NHS).
- If favourable results are found, the potential to implement this screening tool more widely across the NHS workforce and to other high-risk worker groups will lead to improvements in the early detection and management of workers’ hand dermatitis, and such favourable outcomes will have wider benefits to the NHS.

## Introduction

Occupational skin disease is one of the most common work-related health problems, and occupational contact dermatitis is the most common type of occupational skin disease, particularly among healthcare workers. In the UK, hand dermatitis is a commonly recognized occupational skin disease, affecting an estimated 84 000 individuals whose condition is caused or aggravated by their work. It is especially common among healthcare workers [1–3]. The 1-year prevalence of self-reported hand dermatitis in healthcare workers in Sweden (9051 workers) and in Norway (2274 workers) was estimated to be 21%. This compares with <10% in the general population [4]. Once an individual has developed irritant hand dermatitis, the prognosis is generally poor and the disease is often detected late in its progression. In a 15-year follow-up study of a Swedish general population sample, about a third of those with hand dermatitis needed ongoing medical treatment and 5% experienced long periods of sickness absence, loss or change of job, or retirement due to ill health, as well as adverse economic implications [5]. Affected individuals may also experience negative psychosocial consequences, e.g. sleep disturbance and interference with leisure activities [6,7].

There is a statutory responsibility under the Control of Substances Hazardous to Health (COSHH) Regulations 2002 for employers to carry out skin surveillance in workers exposed to a known hazard in the workplace and where there is potential for significant exposure [8]. The reality of delivering health surveillance for hand dermatitis is challenging for employers, workers and healthcare professionals due to logistic and economic issues. Thus, there is a necessity to find a way to efficiently deliver a surveillance programme. Novel digital skin assessment approaches are increasingly being developed and tested into clinical practice (including occupational health) to improve early detection and monitoring of dermatitis. It refers to the use of technology—such as imaging, mobile apps and AI algorithms—along with questionnaires to evaluate skin conditions remotely or in clinical settings [9,10].

This scoping review aimed to identify which methods of digital skin assessment are effective in a workplace setting and comprised a series of questions (i) What is the evidence for effective digital skin assessment methods for workplace skin surveillance? (ii) What is the context and mode of delivering digital skin assessment methods for workplace skin surveillance? (iii) How effective are digital skin assessment methods for the detection of hand dermatitis among at-risk worker groups such as healthcare workers, hairdressers and construction workers etc.

## Methods

The scoping review was developed using the Preferred Reporting Items for Systematic Reviews and Meta-analysis Protocols (PRISMA-Scoping Review) and based on the PICO framework set out in Table 1. Population: (Employe* OR worker* OR staff OR labour) AND Intervention: Skin surveillance AND Comparator: Any comparator AND Outcome: Hand dermatitis OR eczema.

**Table 1:** Inclusion and exclusion criteria for each study

Inclusion	Exclusion
**Participant demographics**	
Involving employees aged 16 years and older (no upper limit)	Involving persons aged under 16 years Involving unemployed persons
**Workplace interventions**	
Evaluating digital surveillance programme related to skin conditions in the workplace Digital interventions delivered by individuals from one or more disciplines from either clinical or non-clinical backgrounds Digital interventions delivered by public or private companies Digital interventions with or without a comparator group	Interventions that have not evaluated either clinical or cost effectiveness
**Outcomes**	
Reporting characteristics of digital surveillance programmes related to skin conditions in the workplace. Reporting clinical effectiveness outcomes based on health outcomes, including (but not limited to) skin disorders diagnosis and hand dermatitis Reporting cost-effectiveness outcomes of surveillance programmes related to skin conditions in the workplace	Reporting no characteristics of surveillance programmes related to skin conditions in the workplace. Not reporting either clinical or cost-effectiveness outcomes of surveillance programme related to skin conditions in the workplace
**Study design**	
Quantitative studies using different methodological designs, e.g. cluster and individually randomized RCTs, case–control studies, cohort studies, observational studies. Qualitative studies using different designs and methods (one-to-one interview/focus groups/field work)	Case studies
**Source of publication**	
Published journals of full-text papers	Abstracts only or conference proceedings

The search strategy was developed and refined in consultation with a library information specialist from King’s College London. The search strategies devised used medical subject headings (MeSH) where available. Additionally, we identified search terms from indexing terms of pre-identified relevant studies, with subsequent expansion of the search terms using relevant synonyms.

MEDLINE, CINAHL, EMBASE and Cochrane Work databases were searched in December 2024. Limits were applied, e.g. excluding papers not in English and restricting inclusion to publications within the window period of interest (2015–24). Database searching used standard functionality, i.e. Boolean operators, truncation. A review of references of the included papers was also undertaken to identify additional papers. The full-search strategy used in MEDLINE and modified for use in other databases can be found in Supplementary material (available at *Occupational Medicine* Online). Grey literature was sourced from professional contacts including Health and Safety Executive (HSE), Society of Occupational Medicine and Faculty of Occupational Medicine.

The protocol was registered with the Open Science Framework https://doi.org/10.17605/OSF.IO/EBCMT. In keeping with scoping review methodology, the purpose of this scoping review was to scope the body of published evidence, and unlike systematic reviews, no formal critical appraisal of the evidence (i.e. assessing quality and risk of bias) of the included studies was performed, although we have included brief details on these points in the discussion where details are available.

Following the PRISMA flowchart, duplicates from the database searching were first removed before the two reviewers (F.F. and I.A.) independently extracted the data and screened titles and abstracts (Stage 1) for inclusion and exclusion criteria, with individual outcome assessments extracted and documented in a separate spreadsheet. Studies assessed as being potentially eligible for inclusion were taken forward to the full-text review (Stage 2), where studies were assessed for inclusion based on the pre-defined inclusion criteria. Where there was a disagreement at either Stage 1 or Stage 2, the two reviewers met to discuss and gain consensus and a third reviewer (K.A.) was available to make the final decision where agreement could not be reached.

Completion of Stage 2 determined the final list of included studies for data extraction.

An electronic data-charting spreadsheet with relevant variables was developed by the study team to extract and record relevant data of interest. Domains included study objectives/aim, study design, description of the study population and intervention, recruitment and referral pathways into the study, setting and mode of delivering the intervention, time/duration of the intervention, comparator, description of measurement tools, description of health and non-health (economic) outcomes, reported barriers and facilitators to intervention delivery, acceptability, effect sizes, statement of cost-effectiveness and overall relevance with our planned interventional study.

## Results

**Figure 1:**
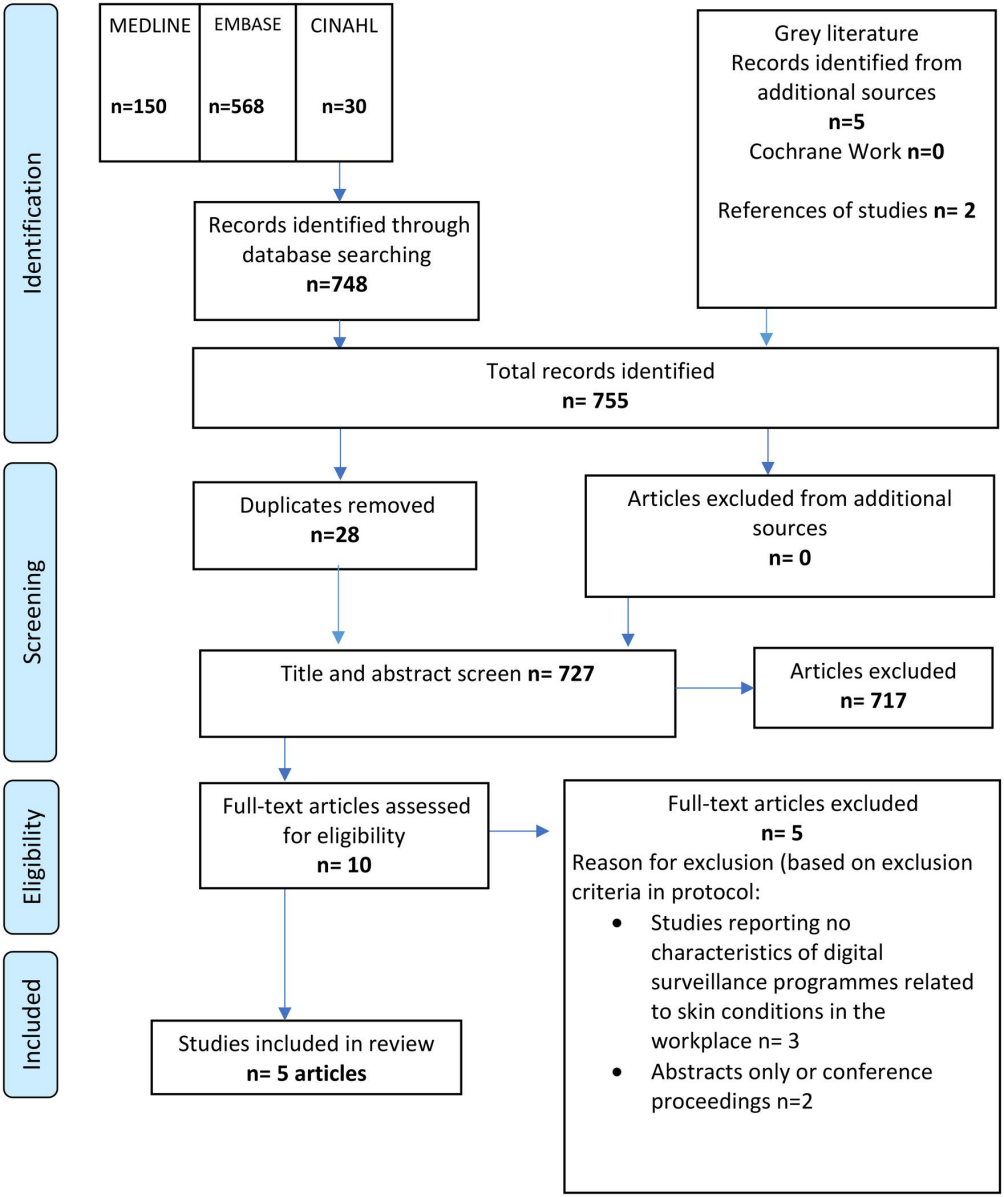
PRISMA flowchart.

Database searching identified 748 studies and 5 sources of grey literature of potential relevance. Twenty-eight duplicate studies were removed. Seven hundred and twenty-seven papers were screened for inclusion based on the title and abstract review (Stage 1), with 717 papers excluded and 10 papers taken forward to the full-text review (Stage 2). Five studies were assessed as meeting the inclusion criteria and were included in the final list of included studies, with the reason for excluding the other five papers outlined in the full PRIMSA flowchart (Figure 1).

Various self-assessment skin surveillance tools and approaches have been developed and evaluated, with positive versus negative predictive values (PPV/NPV) and sensitivity versus specificity measures used to evaluate their diagnostic accuracy: UK (*n* = 2), the Netherlands (*n* = 1), Canada (*n* = 1) and Korea (*n* = 1). At-risk populations investigated include nurses, construction workers, healthcare employees and military personnel, respectively [11–15]. Different methods ranged from (i) single-item self-assessment screening of current hand dermatitis supplemented with standardized hand photographs (‘selfies’) collected via smartphone techniques with remote dermatology nurse or dermatologist evaluation using Coenraads’ photographic validated guide [11,12], (ii) a Rapid Hand Dermatitis Screening Tool administered in clinical settings with digital hand photos collected for dermatological assessment [14] and (iii) brief clinical history taking (demographics, symptoms) for remote teledermatology assessment with comparisons by face-to-face examinations [13,15].

One UK study and its embedded study-within-a-study examined the predictive value of a single-item screening question—‘In your opinion, do you currently have hand/wrist dermatitis?’ (yes/no/unsure)—followed by a location prompt (left/right hand and palm/dorsal) among intensive care and first-year student nurses [12,16]. Standardized photographs were independently assessed (clear → very severe) by a specialty-trained dermatology research nurse and study dermatologists. The study showed this screening method has a high NPV (91%; 95% CI 89–93), but a low PPV (39%; 95% CI 34–45), with high specificity (86%; 95% CI 84–88) but a sensitivity of only 52% (95% CI 46–59). This indicates that the screening question underestimates rather than overestimates the point prevalence of hand dermatitis and has difficulty in detecting very early disease. Nevertheless, the tool was practicable to deliver and reliable for screening out ‘clear’ (no hand dermatitis) cases. While the findings were favourable, they cannot be generalized to other nursing and non-nursing worker groups.

A similar Canadian study investigated the validity of the workplace Hand Dermatitis Screening Tool among hospital workers who are at high risk of hand dermatitis due to wet work [14,17]. The tool collects data on (i) demographics and workplace characteristics, (ii) wet work exposure, glove use and frequency of handwashing, (iii) history of skin disease, (iv) three screening skin condition categories (a) normal (clear skin, no redness and no dryness), (b) mild (small areas of the hands have slight redness and/or dryness) and (c) moderate/severe (large areas of the hands have slight redness and/or dryness, or small areas of the hands have severe redness and/or dryness, or areas of the hands have severe redness, dryness, scaling, fissures, crusts or scabs, vesicles and/or papules) and feasibility feedback. Validity testing focused on the sensitivity of the tool by comparing self-screening and nurse screening results with occupational dermatologist screening results using assessments of digital hand photographs. A photographic guide aided the occupational health nurse (OHN) or participants in their screening assessment classification of hand dermatitis. Agreement between workplace screening (either by OHNs or through self-screening) and dermatologist photo assessments (as reference) was fair (weighted *κ* = 0.27) and similar for OHN screening (*κ* = 0.23) and self-screening (*κ* = 0.30). The study found the main source of disagreement between workplace screening (OHN or self-screening) and dermatologist screening lay in the differentiation between the classification of ‘normal’ and ‘mild’ dermatitis. The feasibility for using the tool was assessed as high; assessments took <2 minutes; and the perceived usefulness was high among OHN (95.5%) and workers (77.9%), though cost-effectiveness was not measured.

A study in 2017 of construction workers in the Netherlands investigated four methods against an expert panel involving dermatologists plus an occupational physician. These included: (i) self-reported questionnaires items, (ii) self-reported photo-based items with illustrative severity levels, (iii) occupational physician assessments (pre/post a 1-day dermatology training) and (iv) the expert panel as reference [13]. This study found that there was low agreement between the reference standard and self-report and occupational physician diagnoses, though physician agreement improved after training; underdiagnosis of mild dermatitis persisted.

Furthermore, the single self-reported question about fissures performed comparably well to the full questionnaire for predicting mild dermatitis (PPV: 83%, NPV: 48%). For severe dermatitis, the PPV of the fissure question alone was 38 versus 43% for self-reported dermatitis; NPVs were similar (84 versus 78%). Additionally, the authors reported that the tool, which was originally validated for use in nurses, was less transferable to industrial workers, likely due to a difference in symptom perceptions and reporting behaviours.

In a Korean army personnel study, remote consultations using smartphone digital photographs (taken by a paramedic without dermatology training to reflect real-life conditions) and multimedia messaging (text) service to convey clinical information (age, the duration of symptoms, significant medical history and/or any concomitant medication) were compared to in-person dermatology assessments [15]. The mean overall agreement among three teledermatologists was 83% (κ = 0.70 across the three most common diagnostic categories). The pooled sensitivity/specificity were: eczema (78/93.1%), viral warts (88/99.6%) and fungal infections (61/98.1%) [13].

None of the included studies in this scoping review reported on qualitative data from participants or clinicians’ experiences of using screening tools.

## Discussion

This scoping review found a limited number of papers describing different, albeit comparable, digital self-assessment skin surveillance tools. Overall, the evidence shows that such tools can reliably and efficiently rule out clear (no dermatitis) cases in some settings, as shown by the high NPV, but at times underestimate early/mild disease and show limited PPV [12–15]. Additionally, other practical challenges associated with implementing self-screening tools were identified and include the potential for workers to deny symptoms altogether, the influence of convenience sampling and self-selection bias within a research study design and the unreliable use of ‘standardised’ methods for collecting digital images. Whilst some of the included studies assessed the inter-rater reliability of assessments (i.e. concordance across different assessors), there would also be benefit in assessing the intra-rater reliability of assessments to establish the degree of consistency in assessment outcomes with the same assessor (e.g. clinician) viewing the same image, or in the case of patients, their own hands multiple times. The benefits of this were also recommended by Nichol et al. [14]. The potential limitations of these included studies also relate to their wider generalizability of findings to other at-risk worker groups, so caution is warranted in this regard.

It should be noted that one area of disagreement in the studies is between the ‘normal’ and ‘pathological’ screening categories. The milder pathological categories used in the studies refer to small patches on the hands showing slight redness and/or dryness, which are much harder to digitally assess with confidence compared to the moderate/severe category, where larger areas or more intense redness/dryness are present. Some health professionals may apply stricter criteria when judging dermatitis, considering very small patches of redness or minimal skin changes as normal, while dermatologists are more inclined to classify these as pathological. This difficulty is compounded by the absence of standardized definitions in the existing literature for what qualifies as mild hand dermatitis and by the fact that disease severity represents a continuum rather than clearly defined categories [18].

Collectively, these consistent findings provide strong evidence that the use of self-assessment screening tools for skin diseases, when combined with remote back-up dermatological assessments using digital images collected on smartphones, are clinically reliable and simple and cost-effective to implement in workplace settings. Moreover, given the diagnostic concordance with the self-assessment method for screening for skin disease is consummate with the gold standard (face-to-face assessments by a dermatologists) and is also more superior to that of clinicians who are not suitably trained or specialized in occupational dermatology, there is justification to undertake a pragmatic study to further assess this digital hand dermatitis screening method involving a wider range of workers groups. If favourable and confirmatory results are observed, then this will provide important research evidence to support recommendations to implement such a tool as part of policy and practice reforms to improve wider workplace skin surveillance programmes across the National Health Service (NHS) workforce and to other high-risk worker groups. Such reforms will then lead to improvements in the early detection and management of workers’ hand dermatitis and will have wider benefits to the NHS.

This is the first published review to assess the scope of available published evidence of different digital methods for screening for hand dermatitis in the workplace context.

Several methodological weaknesses are acknowledged. First, we decided to restrict the search period to studies published in the last 10 years on the basis that we theorized that technological advances in digital skin surveillance methods were more likely to be most applicable during this time period, and so it is possible that the scoping review did not take into consideration earlier effective methods, which could be adopted for use within the modern workplace setting. Furthermore, the scoping review focused on exploring interventions delivered within a workplace setting only, and so it is possible that other non-workplace interventions (e.g. community or educational environments) may have offered important findings, which could be extrapolated and tested in the workplace context. Additionally, we acknowledge the potential impact of selection bias when undertaking this scoping review, although this was minimized because two independent reviewers were involved in the two-stage screening process, with a third arbitrator available if necessary. We acknowledge that while the included papers did not assess and consider the potential use and benefits of artificial intelligence in workplace skin surveillance, we postulate that this fast-paced, evolving field of occupational dermatology will likely have important practical implications for future digital skin surveillance research.

Findings from this scoping review provide reassurance that our follow-up pragmatic study to test the effectiveness of a brief single-item questionnaire with follow-up of those reporting possible hand dermatitis using teledermatology (selfie-taken with a mobile phone camera) should be taken forward in order to establish whether this new digital skin surveillance tool is feasible, acceptable and effective as a screening tool in a workplace surveillance programme in the NHS in the UK. Crucially, if the results of this pragmatic study are favourable, then this is likely to have significant implications for policy and practice, particularly in terms of revolutionizing the early screening, detection and management of hand dermatitis among clinical and non-clinical workers and this is likely to be generalizable to other industries.

## Supplementary Material

kqag028_Supplementary_Data
