# Supplementary material for: Investigating the evidence for effective digital skin surveillance methods
Source: Occup Med (Lond). 2026 Jun 2;76(4):230–6. doi: 10.1093/occmed/kqag028 (PMC13412379; doi:10.1093/occmed/kqag028)
Supplement: kqag028_Supplementary_Data [file kqag028_supplementary_data.zip › 25-OP-150.R1 Supplementary_material_(MEDLINE_search_strategy).doc]

—————————————————————————————————————

Database: Ovid MEDLINE(R) ALL <1946 to September 02, 2025>

Search Strategy:

1 exp digital health technology/ or digital*.mp. or exp digital health/ or exp digital

imaging/ or exp "digital imaging and communications in medicine"/ (268376)

2 exp telemedicine/ or exp telehealth/ (53986)

3 tele*.mp. or exp teleconsultation/ (279810)

4 exp health program/ or program*.mp. (1376802)

5 online.mp. or exp online analysis/ or exp online system/ or exp online monitoring/

(297463)

6 exp electronic health record/ or electroni*.mp. or exp electronic medical record/

(517836)

7 film.mp. or exp film/ or exp "photography and film"/ (176489)

8 photo*.mp. (1017464)

9 exp camera/ or camera*.mp. (67102)

10 exp video surveillance/ or exp video consultation/ or exp video interview/ or

video*.mp. (257448)

11 Ehealth.mp. (9206)

12 exp mobile application/ or mhealth.mp. or exp mobile phone/ (45422)

13 "tele referral".mp. (5)

14 exp virtual assistant/ or virtual.mp. (123852)

15 distan*.mp. (548413)

16 assess*.mp. (4902463)

17 evaluation study/ or exp "evaluation and follow up"/ or evaluat*.mp. (5482408)

18 determin*.mp. (4580250)

19 estimat*.mp. (1613871)

20 screen*.mp. (1228374)

21 examin*.mp. (3808536)

22 inspect*.mp. (67795)

23 interven*.mp. (1740980)

24 surveillance.mp. or exp monitoring/ (332109)

25 exp monitor/ or monitor*.mp. or exp patient monitor/ (1366030)

26 counsel$ing.mp. or exp counseling/ (140055)

27 exp e-counseling/ (81)

28 work*.mp. (2453320)

29 exp workplace/ (33419)

30 compan*.mp. (96252)

31 enterpri*.mp. (20265)

32 exp organization/ or organization.mp. or exp health care organization/ (1291980)

33 employee.mp. or employee/ (39843)

34 exp staff nurse/ or exp medical staff/ or exp staff training/ or exp cleaning staff/ or

exp frontline staff/ or exp university staff/ or exp nursing staff/ or exp dental staff/ or

exp correctional staff/ or staff.mp. or exp hotel staff/ or exp staff/ (339458)

35 exp occupation/ or occupation*.mp. (403990)

36 job.mp. or exp work/ (170914)

37 "hand derma*".mp. (8615)

38 "hand eczema*".mp. (1523)

39 "hand erythema*".mp. (9)

40 "occupational derma*".mp. (1982)

41 "irritant derma*".mp. (465)

42 "contact derma*".mp. (16846)

43 "remote consulta*".mp. (7172)

44 1 or 2 or 3 or 4 or 5 or 6 or 7 or 8 or 9 or 10 or 11 or 12 or 13 or 14 or 15 or 43

(4367719)

45 16 or 17 or 18 or 19 or 20 or 21 or 22 or 23 or 24 or 25 or 26 or 27 (16224756)

46 28 or 29 or 30 or 31 or 32 or 33 or 34 or 35 or 36 (4034965)

47 37 or 38 or 39 or 40 or 41 or 42 (26249)

48 44 and 45 and 46 and 47 (385)

49 limit 48 to (english language and yr="2015 -Current") (172)

—————
